# Structural insights into the human niacin receptor HCA2-G_i_ signalling complex

**DOI:** 10.1038/s41467-023-37177-6

**Published:** 2023-03-27

**Authors:** Yang Yang, Hye Jin Kang, Ruogu Gao, Jingjing Wang, Gye Won Han, Jeffrey F. DiBerto, Lijie Wu, Jiahui Tong, Lu Qu, Yiran Wu, Ryan Pileski, Xuemei Li, Xuejun Cai Zhang, Suwen Zhao, Terry Kenakin, Quan Wang, Raymond C. Stevens, Wei Peng, Bryan L. Roth, Zihe Rao, Zhi-Jie Liu

**Affiliations:** 1 iHuman Institute, ShanghaiTech University, Shanghai, 201210 China; 2 National Laboratory of Biomacromolecules, CAS Center for Excellence in Biomacromolecules, Institute of Biophysics, Chinese Academy of Sciences, Beijing, 100101 China; 3 University of Chinese Academy of Sciences, Beijing, 100049 China; 4 Department of Pharmacology, and NIMH Psychoactive Drug Screening Program University of North Carolina Chapel Hill Medical School, Chapel Hill, NC 27514 USA; 5 Department of Biological Sciences, Sungkyunkwan University, Suwon, South Korea; 6 Departments of Biological Sciences and Chemistry, Bridge Institute, University of Southern California, Los Angeles, CA 90089 USA; 7 Innovation Center for Pathogen Research, Guangzhou Laboratory, Guangzhou, 510320 China; 8 Present Address: Department of Obstetrics and Gynecology, Duke University, Durham, NC USA

**Keywords:** Cryoelectron microscopy, X-ray crystallography, G protein-coupled receptors, Permeation and transport

## Abstract

The hydroxycarboxylic acid receptor 2 (HCA2) agonist niacin has been used as treatment for dyslipidemia for several decades albeit with skin flushing as a common side-effect in treated individuals. Extensive efforts have been made to identify HCA2 targeting lipid lowering agents with fewer adverse effects, despite little being known about the molecular basis of HCA2 mediated signalling. Here, we report the cryo-electron microscopy structure of the HCA2-G_i_ signalling complex with the potent agonist MK-6892, along with crystal structures of HCA2 in inactive state. These structures, together with comprehensive pharmacological analysis, reveal the ligand binding mode and activation and signalling mechanisms of HCA2. This study elucidates the structural determinants essential for HCA2 mediated signalling and provides insights into ligand discovery for HCA2 and related receptors.

## Abstract (web summary)

Hydroxyl-carboxylic acid receptor 2 (HCA2) functions as a high-affinity receptor for nicotinic acid (vitamin B3). Here, authors report the cryo-EM structure of the HCA2-G_i_ complex with the agonist MK-6892 and inactive state crystal structures of mutation stabilized HCA2, to describe the mechanism of HCA2 signaling.

## Introduction

G-protein coupled receptors (GPCRs) represent a class of integral membrane proteins that interact with a vast array of neurotransmitters, hormones, odorants, lipids, ions and metabolites^1,2^. HCA2, also known as GPR109A or niacin receptor, is a prototypical metabolite-sensing receptor^3^ and also have long represented the molecular target for the anti-dyslipidemic actions of niacin and the endogenous ligand 3-hydroxy-butyric acid^4–7^, being enriched on adipocytes. Many high-affinity ligand have been developed by academia and industries to mimic niacin’s antilipolytic effect. Compounds, such as MK-1903 and acifran, were developed as selective high affinity HCA2 agonists and were demonstrated to lower free fatty acids in humans^8^. A more recently developed compound, MK-6892 represents one of the most potent HCA2 agonists discovered^9^. In addition to the efforts to develop high-affinity ligand, the field also tried to develop safer drug since niacin use was limited by its well-known side effect, skin flushing. Therefore niacin analogues, such as MK-0354 with reduced flushing profile^9–11^, were generated, also suggesting there may be relationship between β-arrestin signalling and skin flushing effect although more studies are necessary to demonstrate this conclusively^12,13^. While the understanding of HCA2 signalling is important^14^, its activation and signalling mechanisms are still illusive due in part to the lack of elucidation of structure-function relationship for any of the HCA1-3 and relevent receptors (i.e. 5-oxo-ETE receptor, OXER1 or GPR31).

HCA2 is an important receptor to understand since it regulates homeostasis during physiological and pathophysiological conditions implicated in a variety of diseases, including cardiovascular diseases, multiple sclerosis, Parkinson’s disease, Alzheimer’s disease, neurological diseases and colon cancer^3,15–19^. In addition, HCA2 plays crucial role in nutrient sensing and anti-inflammatory effect using various signaling mechanisms^20–24^. Therefore, many developed ligands with high affinity or reduced arrestin signaling would be useful tool to understand the role of HCA2 in those diverse diseases.

In this work, we present the 2.7 Å inactive HCA2 crystal structures and a 3.1 Å cryo-electron microscopy (cryo-EM) HCA2-G_i_ complex structure with the potent compound MK-6892 and antibody fragment ScFv16, together with results from G-protein and β-arrestin signaling functional analysis. This study reveals the active and inactive states of the receptor and illuminates potential mechanisms for HCA2 activation.

## Results

### HCA2 exclusively couples to G_i/o_ family

HCA2 is known to couple to members of the G_i/o_ family of heterotrimeric G proteins^4–7^, but there has not been a comprehensive analysis of its coupling preferences either among members of this family or for non-G_i/o_ proteins. Therefore, we measured the potential of HCA2 to activate 14 different G_α_ subunits representing all four G protein families (G_12/13_, G_i/o_, G_q/11_, and G_s/olf_) using our BRET-based TRUPATH platform^25^. We observed robust coupling of HCA2 to G_i/o_ family members in response to both niacin and MK-6892 activation, but negligible coupling to non-G_i/o_ family proteins (Supplementary Fig. 1a–c). Importantly, the most potent coupler to MK-6892-bound HCA2 was G_i1_, and thus we sought obtainment of this complex for structural determination.

### MK-6892 is one of the most potent HCA2 ligands

Many HCA2 agonists were generated as selective and high affinity ligands and MK-6892 was developed most recent. Our data and others confirmed that MK-6892 is one of the most potent HCA2 agonists either in cAMP G_i_ activation or β-arrestin recruitment^13^ (Supplementary Fig. 1d). The strong arrestin recruitment properties of MK-6892 are interesting since MK-6892, which also has reduced skin flushing^9^, showed strong arrestin activation, although the weak arrestin activation of MK-0354 was postulated to be relevant to reduced skin flushing. This means that the correlation between arrestin signalling and skin flushing requires further research (Supplementary Fig. 1d).

### Structures of HCA2-G_i_ complex and inactive HCA2

**Fig. 1:**
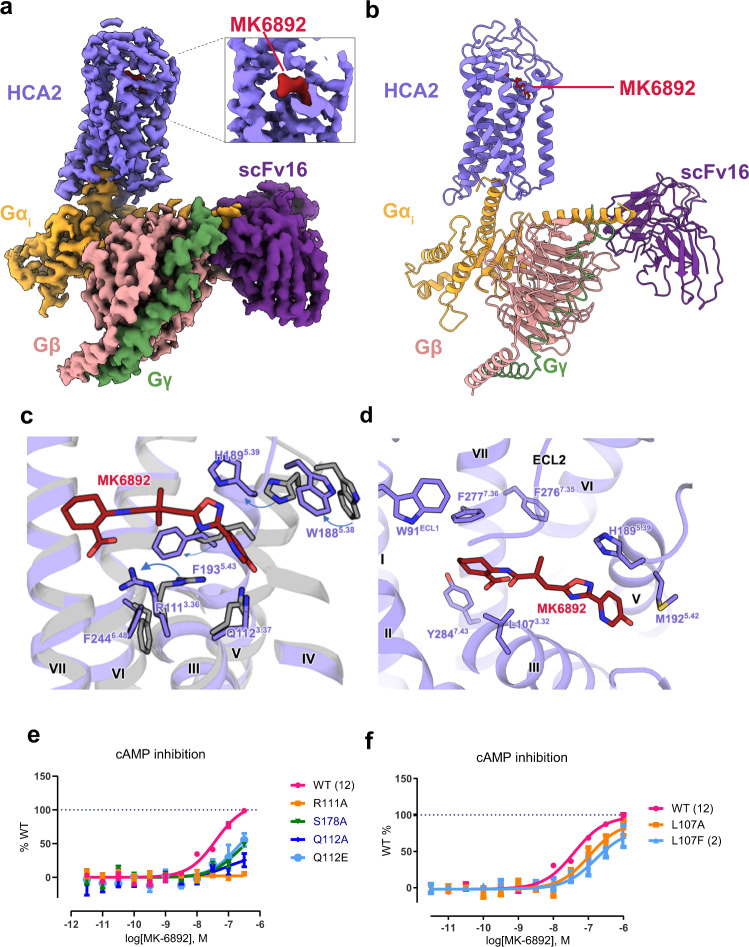
Cryo-EM structure and binding pocket of HCA2-G_i_ complex. **a** Cryo-EM map of HCA2-G_i_ complex colored by subunit (HCA2, light purple; MK-6892, red; heterotrimeric G_i_, orange, pink and green for α, β and γ, respectively; ScFv16, velvet). Box indicates zoomed in view of MK-6892. **b** Determined cryo-EM structure of HCA2-G_i_ complex in model cartoon performance. **c** Zoom in superposition of MK-6892 bound state and inactive state HCA2 structures with the conformation change of the binding pocket and helix V. Several obvious pairs of residue movements are indicated by arrows. MK-6892 bound state in purple and inactive state in grey. **d** MK-6892 bound to HCA2 orthosteric binding pocket interacts with hydrophobic residues. **e** MK-6892 induced G_αi/o_-mediated signalling at WT, R111A, S178A and Q112A/E. **f** MK-6892 induced G_αi/o_-mediated signalling at WT L107A/F.

For cryo-EM studies, the stable complex was successfully constituted by co-expressing engineered HCA2 receptor with N-terminus BRIL fusion, agonist MK-6892, G_i_ (G_αi1β1γ2_) protein and antibody fragment ScFv16^26^. The cryo-EM analysis yielded the HCA2-MK-6892-G_i_-scFv16 (HCA2-G_i_) complex structure at a global resolution of 3.1 Å with most regions of HCA2, MK-6892, G_α_, G_β_, G_γ_ and ScFv16 visible (Fig. 1, Supplementary Fig. 2, Supplementary Tables 1, 2). All transmembrane helices of HCA2 are modeled in the map with the contour level at 3.0 σ (Supplementary Fig. 3a). Agonist MK-6892 is modeled into the orthosteric binding pocket below the extracellular loop 2 (ECL2) based on the EM map (Supplementary Fig. 3b).

In parallel, the crystal structures of mutation induced inactive HCA2, which were expressed in mammalian and insect expression systems, were both solved at 2.7 Å resolution (Supplementary Fig. 4, construct ID 8519 and 3378) and share identical structures with RMSD of Cα atoms below 0.5 Å, with no differences for the bound lipids. The engineered HCA2 molecule is not capable of binding [^3^H]-nicotinic acid based on saturation binding assay or mediating ligand induced signalling and it is likely that one of the thermostabilizing mutations, S287^7.46^V (superscripts denote Ballesteros-Weinstein numbering), locks HCA2 into an inactive state (Supplementary Fig. 4g, Supplementary Table 3).

Comparing the HCA2 receptor in different states, upon activation, the agonist MK-6892 forms close interactions with helix V and ECL2, and initiates substantial conformational changes in HCA2. The helix V converges in extracellular portion about 4.9 Å (main chain) and moves outwards in intracellular region, while helix VI near extracellular region moves inward about 1.9 Å (Supplementary Fig. 5), where MK-6892 binding pocket shrinks with the movement of helix V, as represented by W188^5.38^, H189^5.39^, F193^5.43^ (Fig. 1c). Thus, although there is no obvious movement of TM3 from inactive to active states in HCA2 structures, the side chain of key residue R111^3.36^ moves 6.5 Å towards MK-6892 with a correspondingly large conformational change, which is different from the lysine conformation change in many class A GPCRs such as A2a^27–29^, M2R^30–32^, μOR^33–35^ by residue R^3.50^.

### Agonist MK-6892 recognition in HCA2

In HCA2-G_i_ complex structure, several hydrophobic and hydrophilic interactions are observed between HCA2 and MK-6892 (Fig. 1c, d). The binding pocket is tightly formed by residues from ECL2, helix II, helix III and helix VII, including the hydrophilic interactions between MK-6892 and R111^3.36^, S178^ECL2^, S179^ELC2^ and Q112^3.37^ (Fig. 1c). The carboxylic group of MK-6892 interacts with R111^3.36^ by strong ionic interactions. Our mutagenesis data supports this binding mode, as the R111^3.36^A mutant abolishes MK-6892’s agonist activity (Fig. 1e) while its cell surface expression level is comparable to WT’s one (Supplementary Fig. 6). In a similar manner, the agonist niacin and other tested agonists lose agonist activity probably due to the impaired interaction between their carboxylic acid group or its isostere (tetrazole moiety of MK-0354) with R111^3.36^, which is consistence with previous modelling reports^4,36,37^. In addition, the residue Q112^3.37^ forms a hydrogen bond with the hydroxyl group attached to pyridine ring of MK-6892. This is supported by our mutational study that Q112^3.37^A decreased MK-6892’s potency by 40-fold. (Fig. 1e, Supplementary Table 3).

Interestingly, the Q112^3.37^A mutation showed ligand specific effect since it decreases MK-6892’s activity significantly (Fig. 1e), but doesn’t affect niacin and other tested agonists’ activities to a similar extent (Supplementary Table 4). These results are supported by the structural finding that Q112^3.37^ interacts with the hydroxyl-pyridine ring of MK-6892, but this moiety is absent at other HCA2 ligands explaining why Q112^3.37^A mutation has lesser effect on these HCA2 agonists than on MK-6892 (Fig. 1c, Fig. 4).

The non-polar interactions between MK-6892 and residues L107^3.32^ are supported by our mutational data. The mutation L107^3.32^A or L107^3.32^F decreases the potency of MK-6892 by 3- or 6-fold, respectively, indicating L107^3.32^ interacts with MK-6892 through weak Van der Waals interactions (Fig. 1f). This observation could implicate to structural based drug design for ligands with better interaction with residue L107^3.32^.

To identify the molecular determinants responsible for MK-6892 signalling, we also performed extensive alanine scanning mutagenesis on 38 residues using G_i/o_-mediated cAMP inhibition assay. Strikingly, mutational profile in response to MK-6892 appears quite different from that of niacin. Niacin’s response was diminished more than 10-fold for the 21 out of 38 mutations comparing with WT (Supplementary Fig. 7a,b). The residues which showed ΔΔlog (E_max_/EC_50_)) larger than 1 (the difference between niacin and MK-6892) turned out to be the three residues, Q112^3.37^A, S114^3.39^A and F232^6.36^A, indicating their important role in MK-6892 specific signalling (Supplementary Fig. 7c,d). Apart from the alanine mutations of residues Q112^3.37^ and S114^3.39^ directly influencing the interactions with MK-6892, the F232^6.36^A mutant resulted in the decrease of MK-6892 mediated G protein signaling, in which the side chain of F232^6.36^ tilt about 90° in the active state and altered the interaction between HCA2 receptor and G protein.

### Signalling cascade of HCA2 in δ-branch GPCRs

**Fig. 2:**
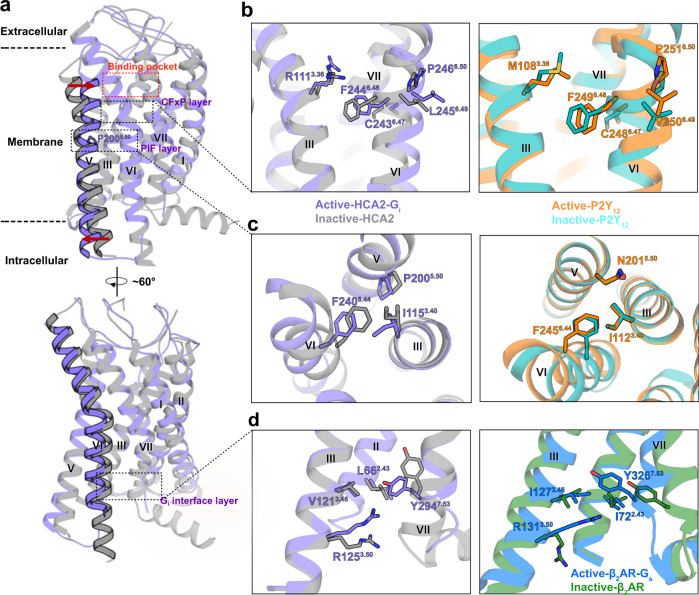
HCA2 activation and conformation changes. **a** Side view of active HCA2 aligned with inactive HCA2, membrane boundaries are marked as dashed lines. Significant local rotation and translation are observed on helix V and VI. **b** Structural comparison of CF(W)xP motif between P2Y_12_ (active PDB: 4PXZ [10.2210/pdb4PXZ/pdb], orange; inactive PDB: 4NTJ [10.2210/pdb4NTJ/pdb], teal) and HCA2 (active: light purple; apo: grey) in active and inactive states, aligned on helix VI. **c** Structural comparison of PIF motif in active and inactive state between HCA2 and P2Y_12_, aligned on helix III. **d** Structural comparison of DPxxY motif in active and inactive state between HCA2 and β_2_-AR (active PDB: 3SN6 [10.2210/pdb3SN6/pdb], blue; inactive PDB: 2RH1 [10.2210/pdb2RH1/pdb], green).

HCA2 belongs to δ-branch in class A GPCR family, which also contains P2Y_1_, P2Y_12_, PAR1/2 and SUCNR1 with solved structures, but less known about their complex with G proteins. Our HCA2 complex shows some hints about δ-branch GPCR activation by investigating the conformation of conserved motifs, including P^5.50^ I^3.40^ F^6.44^, and CF(W)^6.48^xP motifs^38–41^. In HCA2, the activation induced signalling cascade transmits through three layers (Fig. 2). Firstly, in the binding pocket layer, the agonist MK-6892 triggers the side chain of R111^3.36^ flip towards the center of orthorsteric binding pocket, which induces the conformational change of L107^3.32^ and Q112^3.37^ in helix III (Fig. 1c). In the meantime, the movement of F193^5.43^ induces helix V kinks towards the orthosteric pocket as well (Fig. 1c). Secondly, comparing HCA2 structures with active and inactive states P2Y_12_ structures, as expected, the conformations of CF^6.48^ xP of HCA2 structures are very similar to that of the corresponding residues in P2Y_12_, indicating that δ-branch GPCR may have similar activation processes (Fig. 2b). In class A GPCRs, the equivalent residue at position 6.48 is tryptophan as a key component for activation^42^, but in many δ-branch GPCRs, such as HCA2, P2Y_1_ and P2Y_12_, the position 6.48 is occupied by Phe, displaying phenalene movement in active state structures instead of W^6.48^ rotation in other GPCRs. Thus, the synergetic upward movement of F6.48 along with the flipping of R111^3.36^ initiate the activation of HCA2, resembling the ‘twin toggle switch’ reported in CB1^43^. In class A GPCRs, typically the P^5.50^ leads to a kinked helix V in the transducer coupled conformation. While in our active state HCA2 and P2Y_12_ (N^5.50^) structures, helix V showed no kink in the helix (Fig. 2a, c). Thirdly, following the ligand binding induced conformation change, the center motifs undergo flip and translocation in the inner layer of DR^3.50^Y and DPxxY^7.53^ motifs (Fig. 2d). In the active structure of HCA2, the Y294^7.53^ establishes interactions with R125^3.50^, V121^3.46^, and L66^2.43^. In summary, through those interaction network of relatively conserved residues, HCA2 transmits the signal from extracellular part to the intracellular portion.

### The interactions between HCA2 and G_i_

**Fig. 3:**
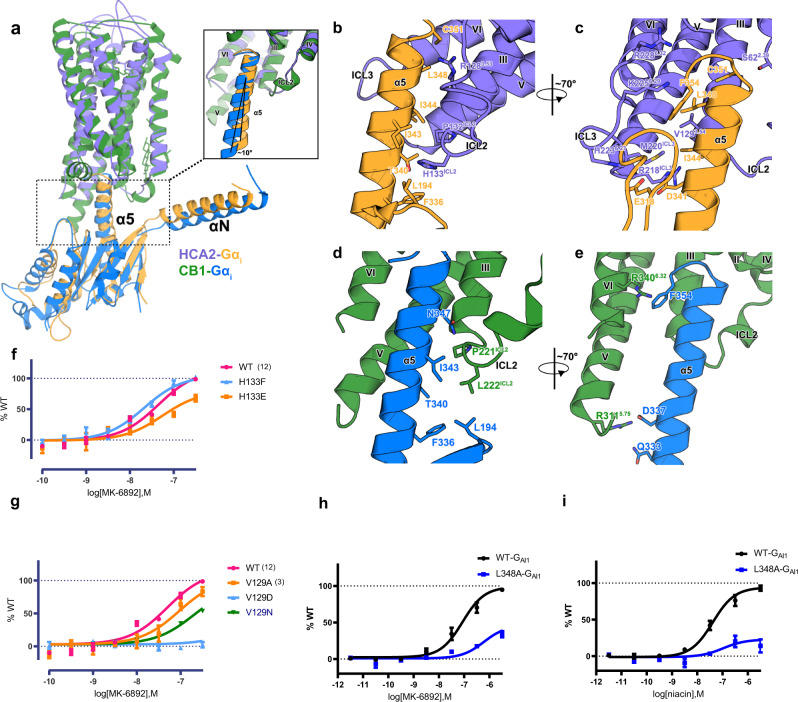
Comparison of HCA2-G_i_ complex with CB1-G_i_ complex in G_i_ protein binding. **a** Relative position of G_αi_ from HCA2-G_i_ complex (orange) and CB1-G_i_ complex (blue), aligned on receptor. The zoomed in view of G_i_ and receptor interface is shown on the right. **b, c** The binding interface of HCA2 (light purple) with G_αi_ (orange) and interacting residues from α5 helix of G_αi_ and adjacent ICL and helices of HCA2. **d, e** The binding interface of CB1 (green) with G_αi_ (blue), in similar views of (**b**) and (**c**). **e, f, g** cAMP inhibition assay of HCA2 interface mutation by MK-6892. **h, i** BRET validation of G_αi1_ interface mutation by MK-6892 and niacin, respectively.

To date, there are several class A GPCR - G_αi/o_ complex structures reported^32,44–50^. Structural comparison of HCA2-G_αi_ with other complex structures are shown in Supplementary Fig. 8 and Fig. 3. Among them, HCA2-G_αi_ is very similar to CB1-G_αi_ with a small conformation shift of α5 and αN in terms of orientations and movements (Fig. 3). In the HCA2-G_i_ complex structure, the main interaction interface is formed by helix III, helix V, helix VI, intracellular loop 2 (ICL2) and ICL3 of the receptor with α5 and αN of the G_αi_ protein (Fig. 3b, c).

The C-terminal helix of G_αi_ (H5) acts as key interface between HCA2 and G_αi_. Specifically, H133^34.51^ of HCA2 forms hydrophobic interactions with α5 of the G_αi1_ subunit, which is also observed in other GPCR-G protein complexes, such as the CB1-G_i_^44,45^ (Fig. 3d, e), MOR-G_i_^49^ and β2AR-G_s_ complexes^51^. Both H133^34.51^ at HCA2 and L222^34.51^ at CB1 are positioned in the hydrophobic pockets (Supplementary Fig. 9a–d). Indeed, our mutagenesis data showed that H133^34.51^E mutation decreased the G_i_ activity of both MK-6892 and niacin, but H133^34.51^F retains the activities of MK-6892 (Fig. 3f) and niacin (Supplementary Fig. 9f) indicating that a polar interaction is not favored in this hydrophobic pocket, rather the aromaticity of histidine is likely to play a role to mediate G_i_ signalling. These results are quite striking since the position of ICL2^34.51^(which corresponds to H133^34.51^ of HCA2) has been stated to play a key role in G_αs_ and G_αq_ coupling, but not G_αi/o_^52–54^. Specially, it was reported that the CB1 mutation L222^34.51^F showed minimal G_i_ activity while increasing basal activity of G_s_^44,45,52^. The corresponding residue mutation at CB2 (P139^34.51^F) also increased the G_s_ activity^53^ which is consistent with the β2AR residue F139 that has an extended hydrophobic interaction with G_s_ protein^51^. Based on our data, we conclude that ICL2^34.51^ plays an important role in G protein coupling.

Additionally, hydrophobic interactions between L348 of α5 and V129^3.54^ of HCA2 are observed by mutational studies, indicating that HCA2 mutation V129^3.54^N decreased the efficacy of niacin and MK-6892 by 50% and V129^3.54^D significantly decreased activities of the two ligands while V129^3.54^A retains the activities, indicating V129^3.54^ is engaged in the interaction of G_αi1_ through hydrophobic interaction (Fig. 3g). The G_αi_ mutation L348A also decrease the activity of MK-6892 or niacin (Fig. 3h, i), indicating that interaction between L348 of α5 and V129^3.54^ of HCA2 play an important role to stabilize the G_αi_/HCA2 interaction by non-polar interaction.

### Conserved key residues for HCA2 ligand recognition

**Fig. 4:**
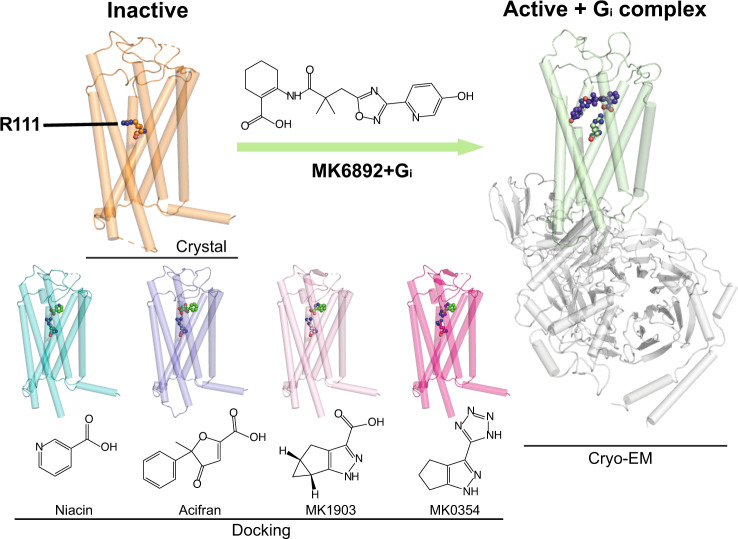
HCA2 key residue with ligands recognition. Key residue and ligands are shown as sticks and spheres. Inactive state HCA2 in orange color, active state cryo-EM structure of HCA2 with MK-6892 and G_i_ complex in green and grey, respectively. Docking structure with niacin in cyan, acifran in purple, MK-1903 in pink, and MK-0354 in magenta.

To understand the molecular basis of interactions between HCA2 and other ligands, we performed induced fit docking of wild type HCA2 with niacin, acifran, MK-1903, and MK-0354 (Fig. 4). Our HCA2-G_i_-MK-6892 complex identified R111^3.36^ in the binding pocket plays a key role to interact with -COOH of MK-6892. The MK-6892 activated complex structure and docking poses of HCA2 with other ligands reveal that R111^3.36^ is the key residue for ligand recognition, which is consistent with mutagenesis data (Supplementary Table 4). The carboxylate moiety of niacin, acifran and MK-1903 form salt bridges or hydrogen bonds with the side chain of residue R111^3.36^, and contributes to a larger binding pocket for these small ligands. R111^3.36^ also forms interaction networks with other residues like Q112^3.37^, E196^5.46^, thus it would have a more structural impact on binding. The residue R251^6.55^ in docking results also show binding with ligands, but as surrounded by F193^5.43^, F255^6.59^ and F276^7.34^, there would be less space for R251^6.55^ to make conformation change to trigger downstream signalling. The docking pose of MK-0354, which has tetrazole moiety, a COOH isostere, resembles that of niacin, it helps explain why MK-0354 is weaker than niacin. The bulkier tetrazole head pushes the guanidino group of R111^3.36^ taking the “down” conformation, which weakens the interaction between them (Supplementary Fig. 10a, b). It is also reported that the stability of tetrazole-amidine complex is lower than that of carboxylate-amidine complex^55^.

Indeed, R111^3.36^ is conserved across all HCA family (Supplementary Fig. 10c). Another hydrophilic interaction partner Q112^3.37^ plays a role of ligand specificity (Supplementary Fig. 10d, Supplementary Table 4). Structural and mutational studies reveal Q112^3.37^ interacts with hydroxyl-group attached to pyridine ring of MK-6892 through hydrogen bond interaction. The fact that this extended chemical moiety of MK-6892 which is absent in other HCA2 ligands suggested Q112^3.37^ may specifically interact with MK-6892.

In this study, we present the HCA2-G_i_ signalling complex and mutation induced inactive state HCA2 structures, providing comprehensive molecular insights into HCA2 ligand selectivity and receptor activation which may share unique activation mechanism with δ-branch members. Thus, structure-based analysis of the significant role of R111^3.36^ as a carboxylate moiety recognition residue, together with Q112^3.37^ as the extension of hydroxyl-group binding site shed light to uncover ligand selectivity of hydrocarboxylic acid receptor. Taken together, this study should accelerate the design of ligands for HCA2 and related receptors both in hydrocarboxylic acid receptor family and δ-branch GPCRs.

## Methods

### Protein engineering for HCA2 crystallography

For HCA2 crystallography we designed a thermostabilized construct mimetic suitable for structural studies with mutations A70^2.47^V and S287^7.46^V on the trans-membrane (TM) regions, a BRIL fusion on the ICL3 loop, and 38 residues truncated from the C-terminal as previously described^56^. These thermostabilizing mutations, fusion insertion and truncations improved protein homogeneity and thermostability. Three disulfide bonds kept the loops less flexible and may have maintained the receptor in a stable state (Supplementary Fig. 4).

A panel of C-terminal truncation, ICL3 replaced with BRIL (Protein Data Bank accession code 1M6T) and two mutants (A70^2.47^V and S287^7.46^V) of HCA2 were designed to obtain a stable protein for crystallization. Two HCA2 constructs were designed for two expression systems: construct 3378 was reconstructed to a pFastBac1 vector for insect cell expression; and construct 8519 was reconstructed to a pTT5 vector for mammalian cell expression. 3378 had an N-terminus Flag-tag and C-terminus His-tag with a TEV cleavage site. 8519 had an N-terminus Flag and His-tag with TEV cleavage site at the beginning of the receptor. Proteins were obtained by 50 mM Tris-HCl pH 7.5, 500 mM NaCl, and 0.5% (w/v) n-dodecyl-b-D-maltopyranoside (DDM) extraction and their expression levels and stability were assayed by size-exclusion chromatography (SEC). During optimization, the construct with 38 truncated residues showed promising stability. In parallel, a panel of ICL3 BRIL insertions was determined in a similar fashion, indicating the insertion between residues 219 and 220 as the most suitable fusion based on SEC examination. Thermostable mutations were designed into the construct by the Quickchange method to improve the temperature melting (Tm) value. Crystals could then be obtained from this construct after screening the crystallization conditions.

### Expression and purification of HCA2 receptor

The two HCA2 constructs 3378 and 8519 were expressed in Spodoptera frugiperda (Sf9) Baculovirus Expression System (Invitrogen) and HEK293 (American Type Culture Collection, ATCC CRL-11268) mammalian expression system using the Bac to Bac and BacMam systems, respectively. The sf9 expression was processed at 27 °C for 48 h. The mammalian expression was processed at 37 °C, 8.0% CO_2_, and 130 r.p.m. for 48 h. Cell pellets were harvested by centrifugation, snap-frozen in liquid Nitrogen and stored at −80 °C. The frozen cell pellets were thawed and resuspended in hypotonic buffer supplemented with a 1:100 (v: v) dilution of mammalian protease inhibitor cocktail (Roche). The membrane was washed repeatedly using a hypotonic buffer with low and high salt, and then suspended in low salt. Before solubilization, purified membranes were incubated with 2 mg/ml iodoacetamide and 100 uM ligand (niacin) and protease inhibitor for 1 h at 4 °C. The HCA2 protein was extracted from the membrane by adding a final concentration of 0.5% (w/v) DDM and 0.1% (w/v) cholesteryl hemisuccinate (CHS) to the membrane solution and was solubilized at 4 °C for 3 h. The supernatant was separated by centrifugation at 160,000 *g* for 30 min, and incubated in TALON IMAC resin at 4 °C overnight. The protein-bound resin was washed with twenty column volumes of 50 mM HEPES, pH 7.5, 0.5 M NaCl, 10% (v/v) glycerol, 0.05% (w/v) DDM, 0.01% (w/v) CHS, and 30 mM imidazole. The protein was eluted with 5 column volumes of 50 mM HEPES, pH 7.5, 0.5 M NaCl, 10% (v/v) glycerol, 0.05% (w/v) DDM, 0.01% (w/v) CHS, 300 mM imidazole, and 200 μM of corresponding ligand. The desalting process to remove imidazole was carried by using a PD MiniTrap G-25 column (GE Healthcare). The HCA2-3378 and 8519 proteins were then treated with His-tagged TEV protease (20 mg per 500 ml of expressed material) and His-tagged PNGase F (20 mg per 500 ml of expressed material) to remove the His-tag and de-glycosylate the receptor. TEV protease, PNGase F, and the cleaved His-tag were removed by reverse binding Ni-NTA superflow resin (Qiagen). The proteins were further concentrated to 20–30 mg/ml using a 100 kDa molecular mass cut-off concentrator (Millipore).

### Crystallization in LCP (liquid cubic phase)

Crystallization trials were performed with the lipid cubic phase method, which is used in many cases of GPCR structure determination. The ratio of protein to lipid (10% (w/w) cholesterol, 90% (w/w) monoolein) was 2:3. The protein-lipid mixture was dispensed in 40 nL drops onto glass sandwich plates and overlaid with 800 nL precipitant solution using a NT8 LCP robot. The crystallization conditions were optimized by screening the pH, PEG400 concentration, salt conditions and adding additives. Crystals grew after 4 days in 100 mM pH5.4 sodium citrate, 60 mM ammonium citrate, 36% PEG400, and 3% Additive 80 (40% PPG), and they reached their full size (80 × 20 × 20 mm^3^) within 3 weeks. Crystals were harvested directly from LCP using 50–100 mm micromounts (MiTeGen) and flash frozen in liquid nitrogen.

### Data collection, structure solution and refinement of crystallography

The X-ray diffraction data of HCA2 were collected at the SPring-8 beam line 41XU, Hyogo, Japan, using a Rayonix MX225HE detector (X-ray wavelength, 0.9162 Å and 1.0000 Å) at 100 K. The crystals were exposed to a 10 mm minibeam for 1 s and 1 u oscillation per frame, and a rastering 11 × 8 mm^2^ minibeam system was used to find the best diffracting parts of single crystals. Most crystals of HCA2 diffracted to 3.3–2.5 Å resolution. Data from individual crystals were processed using XDS^57^ and a complete data set was merged using the data collection strategy option of the program Xscale^58^. The HCA2 structure was solved with Phaser^59^ by molecular replacement (MR) with the structures of Bril and the human P2Y_12_ receptor as the initial search models. The structure was refined to 2.7 Å with good refinement statistics using PHENIX^60^, Buster^61^ and COOT^62^. The 8519 structure was solved by 3378 as a MR search model. Ramachandran statistics of 3378 is 98.71% in favored region and 8519 is 97.45% in favored region, respectively. There are no outliers found in both structures. Model statistics are given in Tables 1 and 2.

**Table 1:** Cryo-EM data collection, refinement and validation statistics of HCA2-G_i_ complex

	HCA2-G_i_ complex (EMD-33241) (PDB 7XK2)
*Data collection and processing*	
Magnification	105k
Voltage (kV)	300
Electron exposure (e–/Å^2^)	60
Defocus range (μm)	−1.0 ~ −2.0
Pixel size (Å)	1.04
Symmetry imposed	C1
Initial particle images (no.)	3685029
Final particle images (no.)	273841
Map resolution (Å)	3.1
FSC threshold	0.143
Map resolution range (Å)	2.9–5.5
*Refinement*	
Initial model used (PDB code)	6N4B
Model resolution (Å)	3.1
FSC threshold	0.5
Map sharpening *B* factor (Å^2^)	−100
Model composition	
Non-hydrogen atoms	8791
Protein residues	1128
Ligands	1
*B* factors (Å^2^)	
Protein	77
Ligand	86
R.m.s. deviations	
Bond lengths (Å)	0.004
Bond angles (°)	0.94
Validation	
MolProbity score	1.72
Clashscore	5.94
Poor rotamers (%)	0.53
Ramachandran plot	
Favored (%)	94.17
Allowed (%)	5.83
Disallowed (%)	0.00

**Table 2:** Data collection and refinement statistics of crystal structures (molecular replacement)

	HCA2 3378 (Insect)	HCA2 8519 (mammalian)
*Data collection*		
Space group	*P*2_1_2_1_2	*P*2_1_2_1_2
Cell dimensions		
*a*, *b*, *c* (Å)	81.01, 82.15, 86.31	80.88, 81.99,85.78
α, β, γ (°)	90, 90, 90	90, 90, 90
Resolution (Å)	47.97–2.70 (2.75 –2.70)^*^	47.8–2.7 (2.80–2.70)^*^
*R*_sym_ or *R*_merge_	0.09 (0.59)	0.12 (0.71)
*I*/σ*I*	10.18 (1.76)	10.91 (2.03)
Completeness (%)	95.76 (92.24)	98.71 (97.84)
Redundancy	5.6 (4.3)	5.9 (4.2)
*Refinement*		
Resolution (Å)	2.70	2.70
No. reflections	23069	16017
*R*_work_/*R*_free_	0.25/0.28	0.25/0.27
No. atoms		
Protein	3168	3100
*B*-factors		
Total	91.54	77.29
Protein	91.21	77.31
R.m.s. deviations		
Bond lengths (Å)	0.003	0.004
Bond angles (°)	0.47	0.62

^*^Number of xtals for 3378 structure is 2 and 8519 structure is 6. ^*^Values in parentheses are for highest-resolution shell.

### Expression and purification of HCA2 with G_i_-protein and antibody complex for cryo-EM

The HCA2 construct for cryo-EM study was containing a BRIL fusion and flag tag epitope at the N terminus, and hexahistidine tag at C terminus. The heterotrimeric G_αi1_β_1γ2_ was constructed same with the previous research^63^, which means G_αi1_ was cloned in pFastbac vector, G_β1_ and G_γ2_ were cloned into another pFastBac Dual vector without hexahistidine tag. The three kinds of plasmids were used for expression in Bac-to-Bac system (Invitrogen). With the baculoviral method, the viruses ratio of HCA2, G_αi1_, G_β1_ and G_γ2_ was optimized and the best one is 1:1:1 in co-expression *sf9* insect cells. The expression was processed at 27 °C for 48 h.

The cells were harvested and lysed in hypotonic buffer with 20 mM HEPES, pH 7.5, 10 mM NaCl, 5 mM MgCl_2_, a pill of cocktail, 100 μM MK-6892 and 10% glycerol. For 1 liter of cell pellets, 5 U of apyrase, 400 μg scFv16 were added and then incubating at room temperature (25 °C) for 2 h. The supernatant was centrifuged at 160,000 *g* for 30 min to collect the precipitants. The precipitants were washed by homogenization and solubilized in 50 mM HEPES, pH 7.5, 300 mM NaCl, 10% glycerol, 0.75% (w/v) lauryl maltose neopentyl glycol (LMNG, Anatrace), 0.15% CHS, and 100 μM MK-6892 for 2 h at 4 °C. The supernatant was isolated by centrifugation at 60,000 *g* for 40 min, and then was incubated with TALON IMAC resin at 4 °C for 6 h. After binding, the complex purification process is the same with crystallography study of HCA2 with final concentration of 100 μM MK-6892 in purification buffer. The elution HCA2-G_i_ complex was concentrated and added 100 μg scFv16 to further stabilize the complex. Then buffer was exchanged to 20 mM HEPES, pH 7.5, 100 mM NaCl, 0.00075% LMNG, 0.00015%CHS, 0.00025% GDN, and 100uM MK-6892. The HCA2–G_i_ complex sample was centrifuged and loaded onto Superdex 200 10/300 GL column and the fractions for the monomeric complex were separated from contaminants and concentrated to about 1.5 mg/ml individually for electron microscopy experiments. The complex samples are further validated with SDS PAGE and Blue-Native PAGE.

### Cryo-EM sample preparation and data acquisition

For grid preparation, complex samples were used either at a concentration of 1.2 mg/ml or 1.6 mg/ml with the same exchanged buffer as described above. The sample (3.5 μl) was applied to glow-discharged holey carbon grid (CryoMatrix Amorphous alloy film R1.2/1.3, 300 mesh) and subsequently vitrified using an FEI Vitrobot Mark IV at 4 °C and 100% humidity. The grids were blotted for 2.5 s at a force of −1 and vitrified by plunge freezing into liquid ethane cooled by liquid nitrogen at −180 °C.

Cryo-EM data were collected on an FEI Titan Krios microscope using a K2 camera positioned post a Gatan GIF quantum energy filter, with a slit width of 20 eV. Micrographs were recorded in super-resolution mode at a magnified physical pixel size of 0.52 Å, with defocus values ranging from −1.0 to −2.0 μm. The total exposure time was 8.0 s and intermediate frames were recorded in an accumulated dose of 60 electrons per Å^2^ and a total of 40 frames per micrograph. Data acquisition was done using SerialEM^64^.

### Image processing and 3D reconstructions

Among all raw cryo-EM stacks, 13,534 micrographs were selected and processed by MotionCor2^65^, on which 5,326,757 particles were picked out using Gautomatch and Ctf estimation was done by Gctf^66^. These particles were fed to a series of 2D and 3D classifications using cryoSPARC^67^ and RELION 3^68^, refined with EMD-0339 as reference, which eventually ended up with a 3.5 Å (gold-standard FSC) EM density map with severe preferred orientation and flattened transmembrane helices. As a remedy, 3,685,029 particles were picked from newly collected 4620 micrographs using template picker of cryoSPARC, then subjected to two cycles 2D classifications in cryoSPARC and 726,362 particles were selected for further 3D process. 4 initial models, generated in Ab-initio Reconstruction of cryoSPARC, were used as template model in Heterogeneous Refinement of cryoSPARC. Finally, 273,841 particles were used to do Homogeneous refinement and Non-Uniform refinement in cryoSPARC, and yield a map of 3.1 Å resolution with sharpened B factor of 132.7. Local resolution variations were estimated by cryoSPARC. More details in Supplementary Fig. 2 Supplementary Fig. 4.

### Model building and refinement

The G_i_ and scFv16 of Cannabinoid Receptor 1-G Protein Complex (PDB: 6N4B) [10.2210/pdb6N4B/pdb] and HCA2 crystal structure in this work were selected as initial models and docked into EM density map using Chimera^69^. The BRIL is not cleaved during the experiment, but the density is missing in the final structure. Then followed real-space refinements using PHENIX and manual adjustments using COOT, aided by secondary structure predictions from Phyre2^70^. Model validation was done by comprehensive validation in PHENIX. More details shown in Table 1.

### Quickchange mutagenesis

Mutations on either the HCA2 Tango construct, which was generated as previously described^71^, or the HCA2 de-tangonized construct (where the V2 tail was deleted from the Tango construct) were generated according to QuickChange II XL-Site-Directed Mutagenesis protocol. In short, PCR was performed using PrimeStarMax DNA polymerase (Clontech) with parental DNA as the HCA2 Tango or HCA2 de-tangonized construct. PCR amplification products were digested with Dpn1 (New England BioLabs) for 1 h in a 37 °C water bath followed by transformation at GC-10 cells (Sigma-Aldrich, G2794). Colonies isolated on an Ampicillin-resistant agar plate that were cultured and prepped using miniprep (QIAprep Spin Miniprep Kit) and midiprep kits (OrigenePowerPrep and HP Plasmid Midiprep, respectively). Sequences were confirmed using a sequencing service (EtonBio or Genescript).

### Cyclic AMP assay

In order to measure HCA2 G_i/o_-mediated cAMP inhibition, CHO cells were transfected with 1 μg of receptor and 1 μg of GloSensor DNA (a luciferase-based cAMP sensor, Promega) using TransIT20/20 transfection reagent (Mirus). The next day, transfected CHO cells were plated in the white 384 well plate with Ham’s F-12 media that is composed of 1% dialyzed FBS media, and 0.5% penicillin-streptomycin. 2-day post-transfection, the cell media was decanted and loaded with 20 μL of drug buffer (20 mM HEPES, 1X HBSS, 0.1% bovine serum albumin (BSA), and 100 μM 3-isobutyl-1-methylxanthine (IBMX), pH7.4) followed by 10 μl of 3X drug solution incubated for 15–20 min. Then 10 μl per well of Luciferin and 20 μM of forskolin (final concentration) was added for another 15–20 min. Luminescence was read on a Spectra Max luminescence reader. Data were analyzed in GraphPad Prism 5.0.

### BRET recruitment assay

To measure G protein recruitment BRET assay, CHO cells were co-transfected in a 1:1:1:1 ratio of G_αi3_-RLuc, G_β3_, GFP2-G_γ9_, and WT or mutant H133E (de-tangoized constructs) respectively. After 24 h, transfected cells were plated in poly-L-lysine coated 96-well white clear bottom cell culture plates with DMEM containing 1% dialyzed FBS, 100 units/ml Penicillin G, and 100 μg/ml Streptomycin at a density of 40,000 cells in 200 μL per well and incubated overnight. The following day, media was removed and cells were washed once with 100 μl of assay buffer (1X HBSS, 20 mM HEPES, pH 7.4, 0.1% BSA). Then 60 μL of assay buffer was loaded per well followed by addition of 10 μL of the RLuc substrate, Coelenterazine 400a (Nanolight) at 5 μM final concentration for 5 mins. Drug stimulation was performed with the addition of 30 μl of 3X drug dilution of MK-6892 or niacin in assay buffer supplemented with 0.01% (w/v) ascorbic acid per well and incubated at RT for another 5 min. Both luminescence (400 nm) and fluorescent GFP2 emission (515 nm) were read for the plate for 1 s per well using Mithras LB940.

GFP2 emission (515 nm) were read for the plate for 1 s per well using Mithras LB940. The ratio of GFP2/RLuc was calculated per well and analyzed using “log (agonist) vs. response” in Graphpad Prism 8 (Graphpad Software Inc., San Diego, CA).

### Radioligand binding assay

The HCA2 radioligand binding assay used [^3^H]-nicotinic acid using Expi293F suspension cells, which express HCA2 receptor or mutants. In brief, Expi293F suspension cells were transfected with WT de-tangonized-HCA2 or mutants constructs for 48 h and the membrane protein was prepared and quantified. For the saturation binding assay, 12.5 μl of 0–100 nM [^3^H]-Nicotinic acid, 25 μl of 50 μg/well membrane protein, and binding buffer (50 mM Tris-HCl, pH 7.40) were mixed to make the final 125 μl reaction, which was then incubated for 1 h at 25 °C. For non-specific binding, 25 μl of 100 μM of nicotinic acid was added. For the competitive binding assay of the WT HCA2 receptor or mutant receptors, 50 μl of membrane protein, 50 μl of 10–30 nM [^3^H]-nicotinic acid, whose concentration was determined from the saturation binding assay, were incubated for 1 h at RT. Then 25 μl of cold-ligand solution was added to the pre-equilibrated membrane-hot ligand mixtures. The reaction was halted using vacuum filtration onto a 0.3% PEI soaked filter mat using a 96-well format harvester and the filter mat was washed three times with cold binding buffer. On top of the dried filter mat, the scintillation cocktail was melted, and radioactivity was measured in a Micro beta counter.

### Surface expression

Cell surface expression was measured using ELISA chemiluminescence. Briefly cells were fixed with 20 μl/well 4% paraformaldehyde for 10 min at room temperature followed by washing with 40 μl/well of phosphate buffered saline (PBS). Then cells were incubated with 20 μl/well 5% BSA (bovine serum albumin) in PBS for 30 min followed by incubation with an anti-FLAG–horseradish peroxidase–conjugated antibody (Sigma-Aldrich, A8592) diluted 1/10,000 for 1 h at room temperature. After washing three times, 20 μl/well Super Signal Enzyme-Linked Immunosorbent Assay Pico Substrate (Sigma-Aldrich) was used for the development of signal. Signal from wild-type (WT HCA2) -transfected cell was used for data normalization.

### Molecular docking

Docking of HCA2 was done with modules in Schrödinger Suites 2018-2. The protein and ligand preparation were done by using Protein Preparation Wizard and LigPrep, respectively. Induced-fit docking was used for ligand docking.

### Reporting summary

Further information on research design is available in the Nature Portfolio Reporting Summary linked to this article.

## Supplementary information

Supplementary Information

Reporting Summary

## Data Availability

The data that support this study are available from the corresponding authors upon request. Cryo-EM maps have been deposited in the Electron Microscopy Data Bank (EMDB) with the accession numbers EMD-33241 (HCA2-G_i_ protein complex). The atomic model has been deposited in the Protein Data Bank (PDB) under accession number 7XK2 (HCA2-G_i_ protein complex). X-ray structures factors have been deposited with the accession numbers 7ZLY (HCA2 3378 (Insect)) and 7ZL9 (HCA2 8519 (mammalian)). Previously published structures referenced can be found under accession code 6N4B (Cannabinoid Receptor 1-G Protein Complex) Source data are provided with this paper.

## Source data

Source Data
