# Supplementary material for: Structural insights into the human niacin receptor HCA2-Gi signalling complex
Source: Nat Commun. 2023 Mar 27;14:1692. doi: 10.1038/s41467-023-37177-6 (PMC10043007; doi:10.1038/s41467-023-37177-6)
Supplement: Supplementary file 1 — Supplementary Information [file 41467_2023_37177_MOESM1_ESM.pdf]

Structural Insights into the Human Niacin Receptor HCA2-G_i Signalling Complex

Yang Yang[1,2,3,9], Hye Jin Kang[4,8,9], Ruogu Gao[2,3,9], Jingjing Wang[1], Gye Won Han[5], Jeffrey F. DiBerto[4], Lijie Wu[1], Jiahui Tong[1], Lu Qu[1], Yiran Wu[1], Ryan Pileski[4,7], Xuemei Li[2,3], Cai Zhang[2,3], Suwen Zhao[1], Terry Kenakin[4], Quan Wang[2], Raymond C. Stevens[1], Wei Peng[6*], Bryan L. Roth[4*], Zihe Rao[2,3*], Zhi-Jie Liu[1,10*]

Affiliations:

[1]iHuman Institute, ShanghaiTech University, Shanghai 201210, China

[2]National Laboratory of Biomacromolecules, CAS Center for Excellence in Biomacromolecules, Institute of Biophysics, Chinese Academy of Sciences, Beijing, 100101, China

[3]University of Chinese Academy of Sciences, Beijing 100049, China

[4]Department of Pharmacology, and NIMH Psychoactive Drug Screening Program University of North Carolina Chapel Hill Medical School, Chapel Hill, North Carolina, 27514, USA

[5]Departments of Biological Sciences and Chemistry, Bridge Institute, University of Southern California, Los Angeles, CA 90089, USA

[6]Innovation Center for Pathogen Research, Guangzhou Laboratory, Guangzhou 510320, China.

[7]Present address: Department of Obstetrics and Gynecology, Duke University, Durham, North Carolina, USA

[8]Department of Biological Sciences, Sungkyunkwan University, Suwon, South Korea

[9]These authors contributed equally

[10]Lead Contact

[*]Correspondence: peng_wei@gzlab.ac.cn; bryan_roth@med.unc.edu; raozh@tsinghua.edu.cn; liuzhj@shanghaitech.edu.cn;

Supplementary Figures

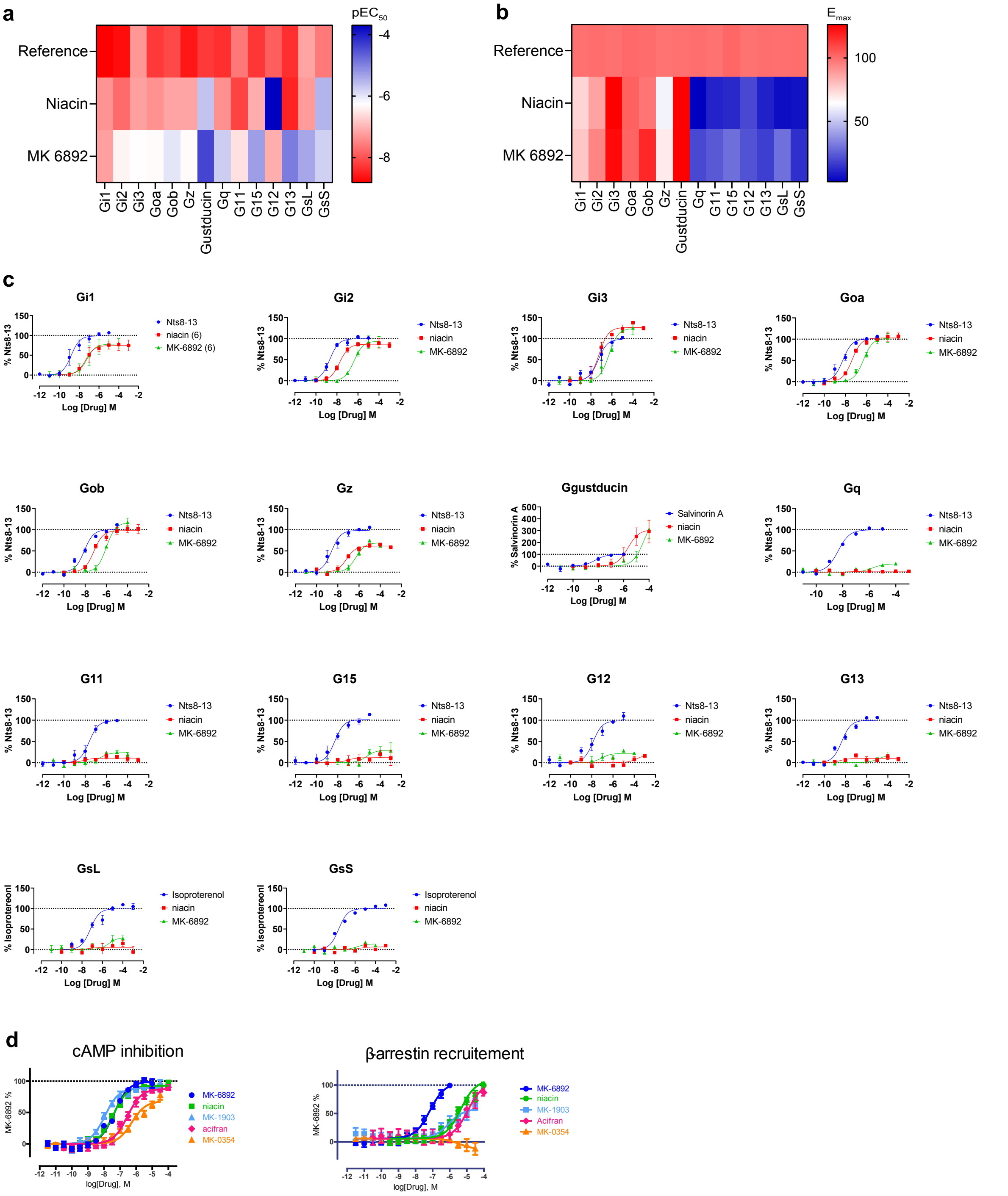

Supplementary Fig.1 HCA2 strongly interacts with $G_{\alpha i/o}$ family member in-vitro (a) Potency heatmap for selected agonists in HCA2 versus a reference agonist for 14 different G_{α} subunits. **(b)** Relative

efficacy heatmap for selected agonists in HCA2 versus a reference agonist for 14 different G_{α} subunits. **(c)** Concentration-response curves for the 14 G_{α} subunits with niacin(red), MK-6892(green) and control (blue) . Data are mean ± s.e.m. of three biologically independent experiments (n = 3) run in duplicates, unless otherwise indicated, in which case the number of biologically independent experiments is indicated in parentheses next to drug. (d) Gαi/o or beta-arrestin mediated signalling at WT HCA2 by MK-6892, niacin, MK-1903, acifran and MK-0354. Data are mean ± s.e.m. of eight biologically independent experiments (n = 8) run in triplicate, unless otherwise indicated, in which case the number of biologically independent experiments is indicated in parentheses next to drug.

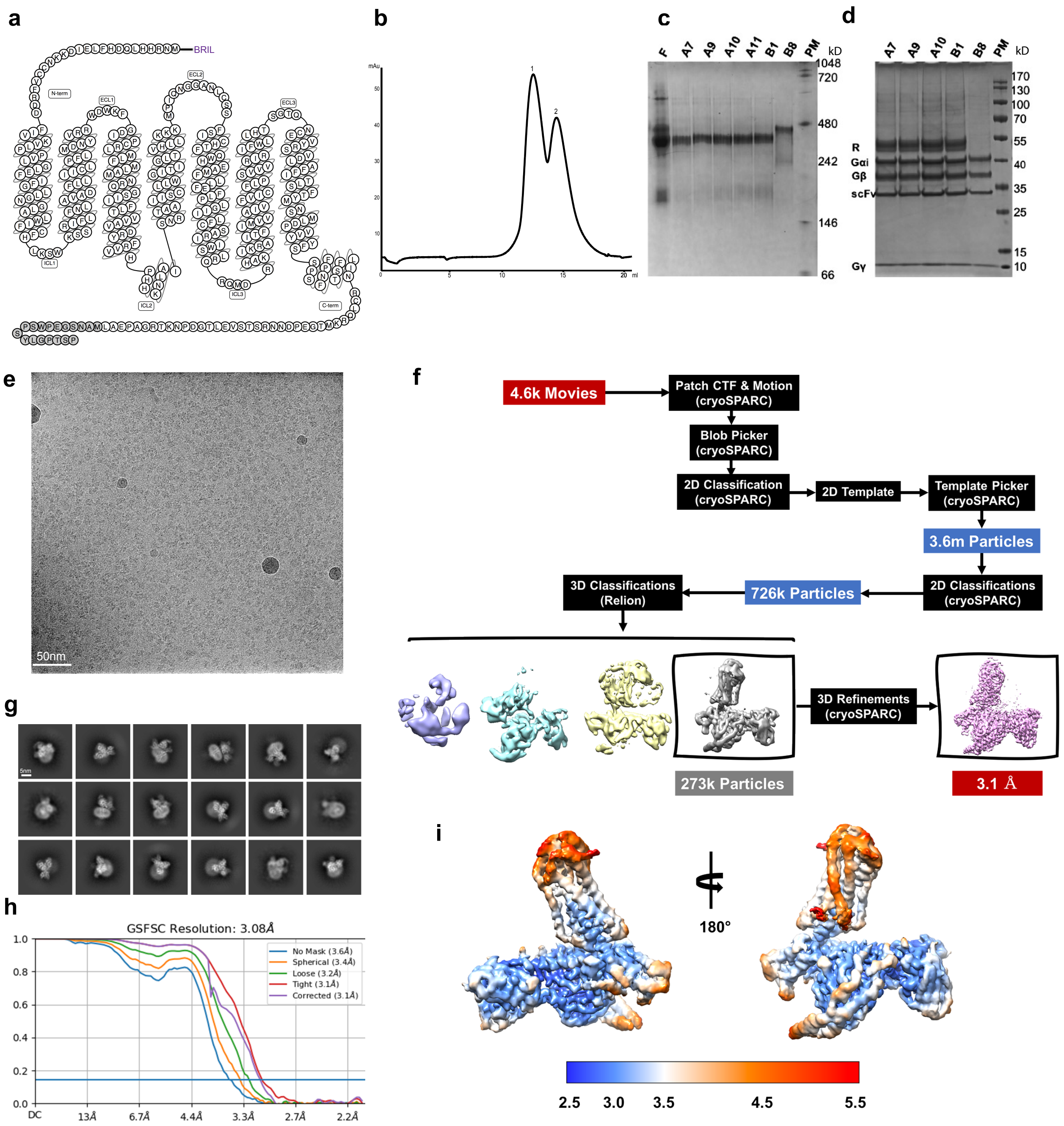

Supplementary Fig.2 Cryo-EM samples and data processing.

(a) Snake plot of the HCA2 construct for Cryo-EM. BRIL is fusion at the N-terminus. Residues in grey circle are truncated. **(b)** Chromatography graph of complex optimization. Peak 1 is the complex and 2 is the G_i-protein with antibody. **(c)** Native page of fast protein liquid chromatography (FPLC) samples. **(d)** SDS Page of FPLC samples. The subunits size is labeled in red color. **(e)** Cryo-EM micrograph of HCA2-

G_i complex. **(f)** Flow chart of cryo-EM data processing. **(g)** Representative 2D averages of HCA2-G_i in 2D classifications. **(h)** FSC chart of HCA2-G_i complex from cryoSPARC refinement. **(i)** Local resolution calculated by cryoSPARC.

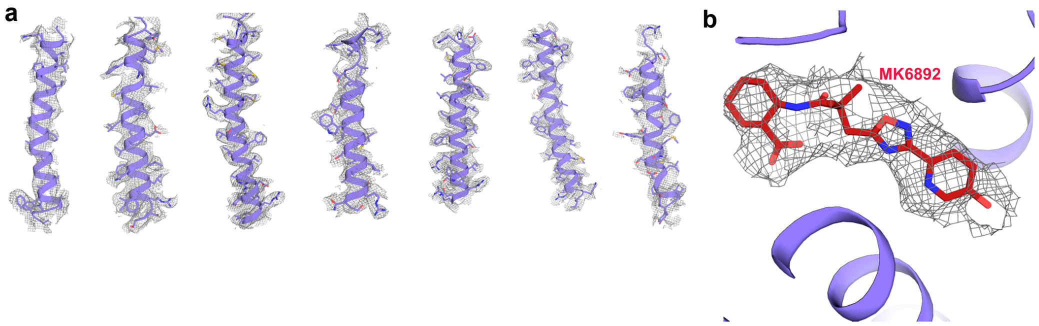

Supplementary Fig.3 Electron microscopy density map of HCA2 related to figure 1.

(a) All transmembrane helices of HCA2 in EM density, map contour level: 3σ. **(b)** MK-6892 in EM density, map contour level: 3σ.

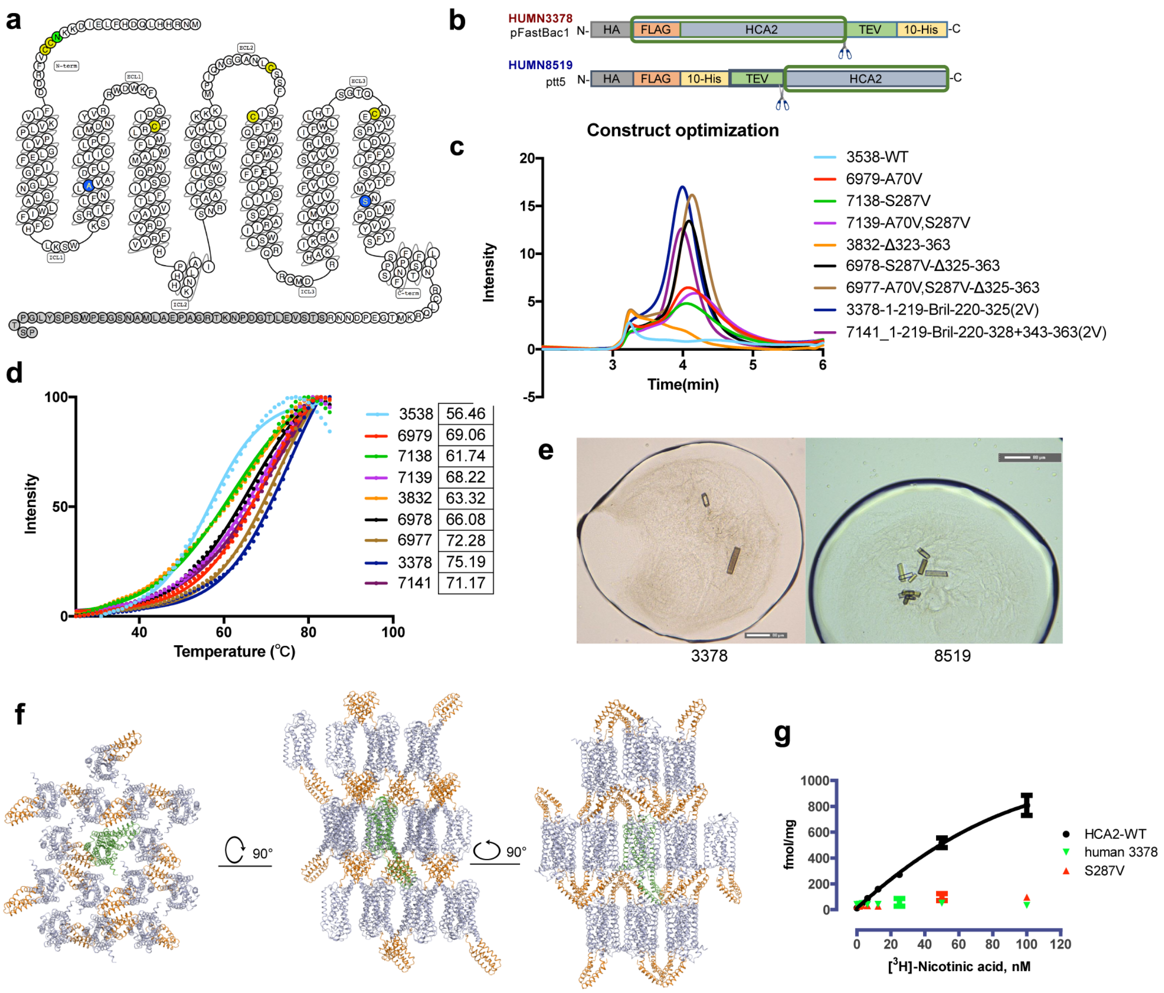

Supplementary Fig.4 Thermostabilized construct and crystal packing of the HCA2 receptor.

(a) Snake plot of the HCA2 thermostabilized construct. Blue circles show the mutations to valines. Grey circles show the truncation region of the C-terminus. Yellow circles indicate three disulfide bonds. Fusion protein BRIL is shown at ICL3. **(b)** Chromatography graph of construct optimization. The truncation, mutation and insertion constructs are validated using their homogeneity peak. **(c)** Thermostabilized CPM graph of the different constructs with the Tm values to the right of the legend. **(d)** The construct design of 3378 and 8519. 3378 had flag-tags in the N-terminus and His-tag in the C-terminus in pFastBac1 vector, which was cut before crystallization. 8519 had tags with Flag- and His-tag in the N-terminus in the pTT5 vector, which were cut before crystallization. The green boxes indicate the purified protein for crystallization. **(e)** Rod-shaped crystals of 3378 and 8519 in LCP. **(f)** Crystal packing of HCA2 in space

group $P2_12_12$. The structure was solved containing the sequence region 12-312, except the 172-173 residues of low densities were not modeled. **(g)** [^{3}H]-nicotinic acid saturation binding assay with HCA2-WT, 3387 or S287V construct. Data are mean ± s.e.m. of three biologically independent experiments (n = 3) run in triplicate, unless otherwise indicated, in which case the number of biologically independent experiments is indicated in parentheses next to construct.

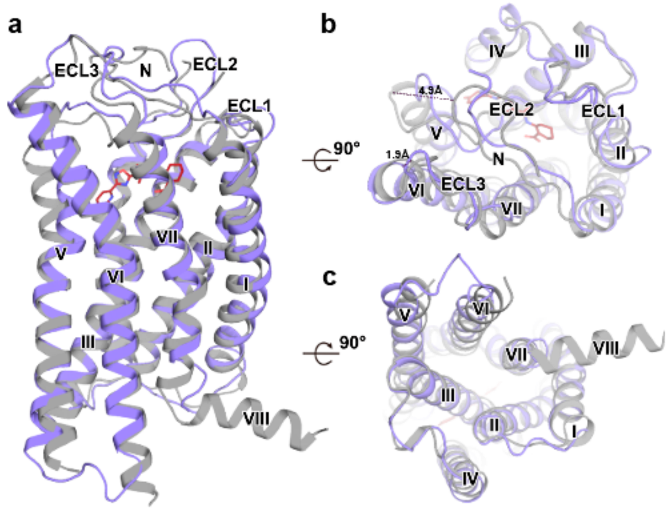

Supplementary Fig.5 Structure comparisons of HCA2 receptor in two states.

(a) Comparison of HCA2 in two states from side view. light purple, active state; gray, inactive. **(b and c)** Top view and bottom view of HCA2 structures. The distances are showed in dash line.

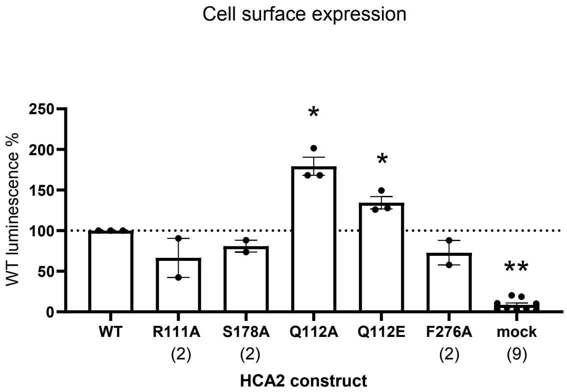

Supplementary Fig.6 Cell surface expression levels of WT and mutants. HEK-293 cells are used to express each construct and the expression levels are measured by ELISA. Data are mean ± s.e.m. of three biologically independent experiments (n = 4) run in triplicate, unless otherwise indicated, in which case the number of biologically independent experiments is indicated in parentheses below the construct name. The mean vaule of each construct was compared to the mean of WT construct using two-sided one-sample T-test. * p<0.5; **<0.0001. Exact p-value of individual without multiple testing correction: p=0.3922 (R111A); p=0.2344 (S178A); p=0.0192 (Q112A); p=0.0448 (Q112E); p=0.3239 (F276A); p<0.0001 (mock).

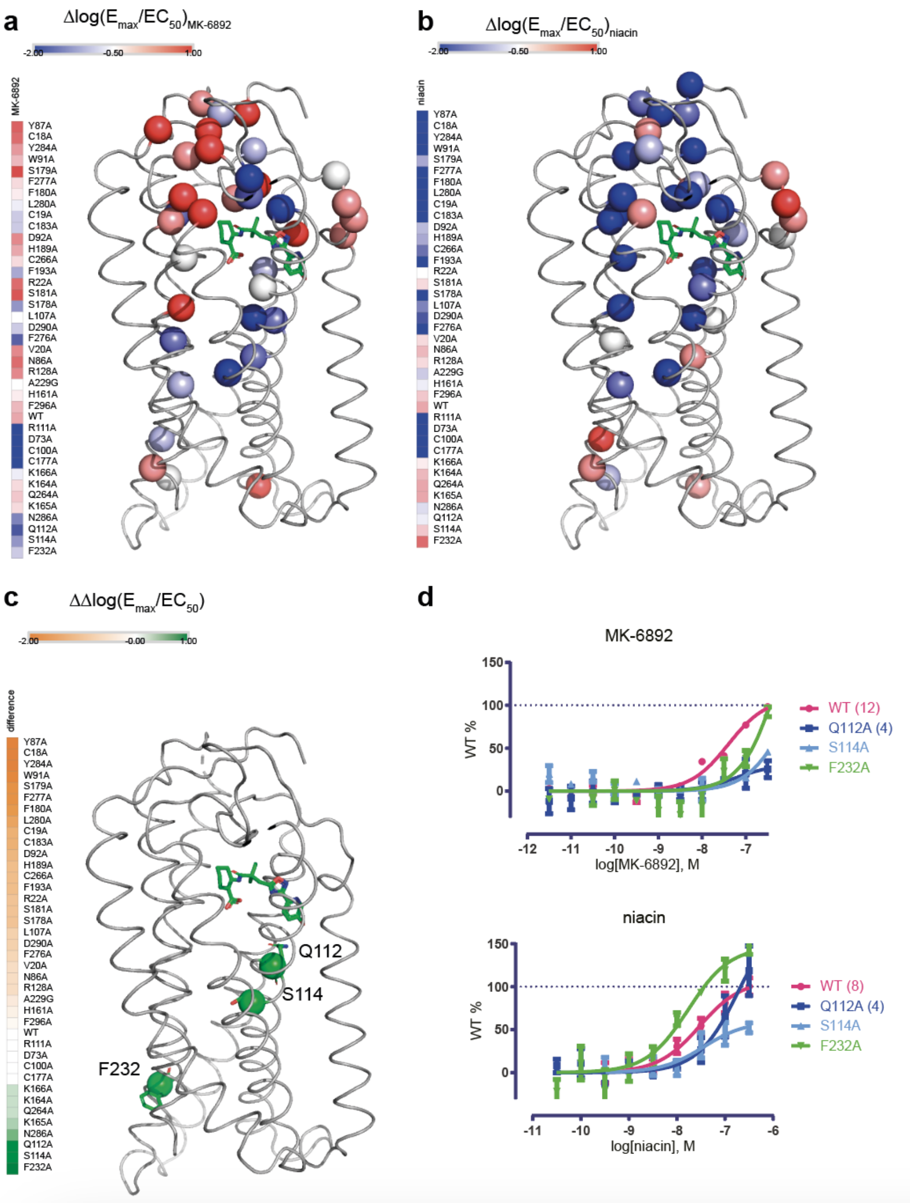

Supplementary Fig.7 Identification of candidates for MK-6892 specific residues.

The Cα atoms, in the color coded depending on the heatmap of $\Delta\log(E_{max}/EC_{50})$, that is $\log(E_{max}/EC_{50})_{mutant}$-$\log(E_{max}/EC_{50})_{wildtype}$ of **(a)** MK-6892 or **(b)** niacin mapped onto the HCA2 structure **(c)** a heatmap $\Delta\Delta\log(E_{max}/EC_{50})$ by subtracting $\Delta\log(E_{max}/EC_{50})$ of niacin from $\Delta\log(E_{max}/EC_{50})$ of MK-6892. The three residues which has the highest value (≥1) are mapped on the Cα atoms onto the HCA2 structure. **(d)** $G_{\alpha i/o}$-mediated signalling at Q112A, S114A and F232 by MK-6892 or niacin. Data are mean ± s.e.m. of three

biologically independent experiments (n = 3) run in triplicate, unless otherwise indicated, in which case the number of biologically independent experiments is indicated in parentheses next to construct.

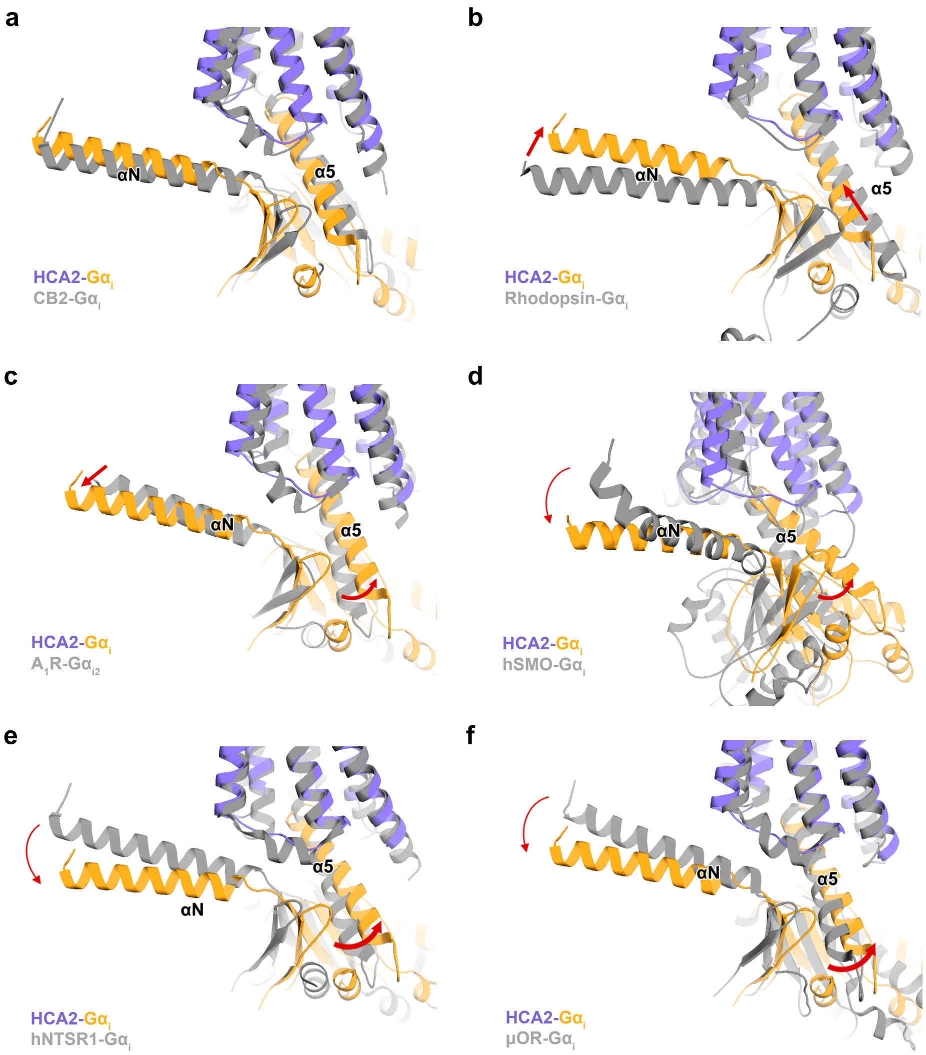

Supplementary Fig.8 Comparison of G-protein orientations in solved $G_{i/o}$ protein complexes.

(a) Comparison of HCA2-G_i complex with CB2-G_i complex. HCA2 in light purple and G_i in orange. CB2 (6KPF) complex in grey. **(b)** Rhodopsin-G_i complex (6CMO) in grey. α5 helix and αN of $G_{\alpha i}$ movement are labeled by red arrows. **(c)** A1 adenosine complex (6D9H). **(d)** M2 complex (6OIK). **(e)** 5HT1B complex (6G79). **(f)** Mu opioid complex (6DDE).

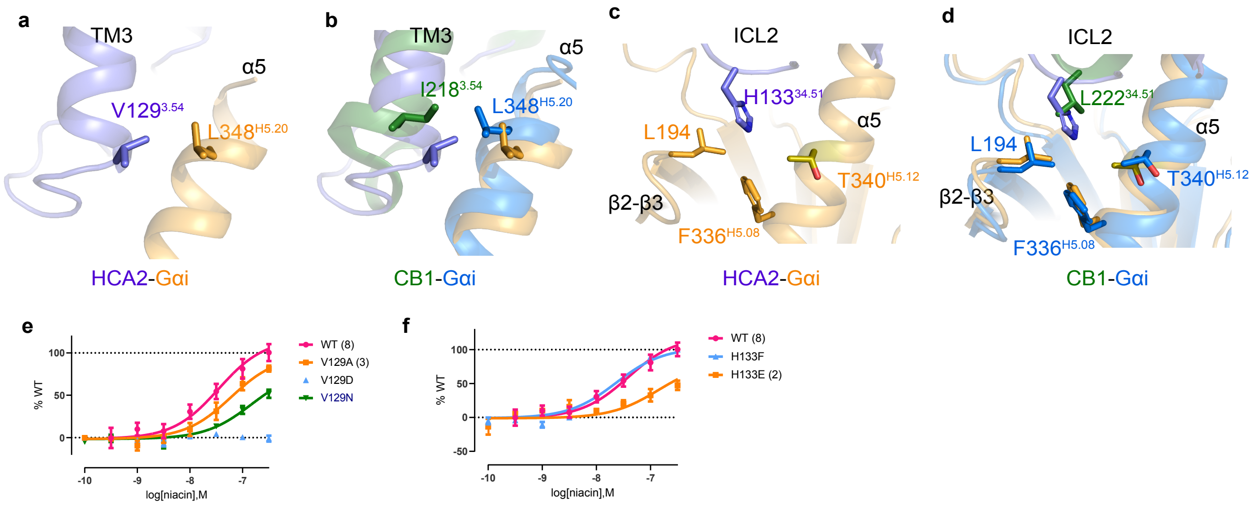

Supplementary Fig.9 Comparison of HCA2-G$_i$ complex with CB1-G$_i$ complex in G$_i$ protein binding. **(a, c)** The close-up view of interaction between HCA2 (light blue) and G$_i$ complex (orange) and **(b, d)** the superimposed structures of HCA2-G$_i$ complex and CB1(green)-Gi1(blue) complexes (PDB ID of CB1-G$_i$ complex: 6N4B). **(e-f)** cAMP inhibition assay of HCA2 interface mutation by niacin. Data are mean ± s.e.m. of five biologically independent experiments (n = 5) run in triplicate, unless otherwise indicated, in which case the number of biologically independent experiments is indicated in parentheses next to construct.

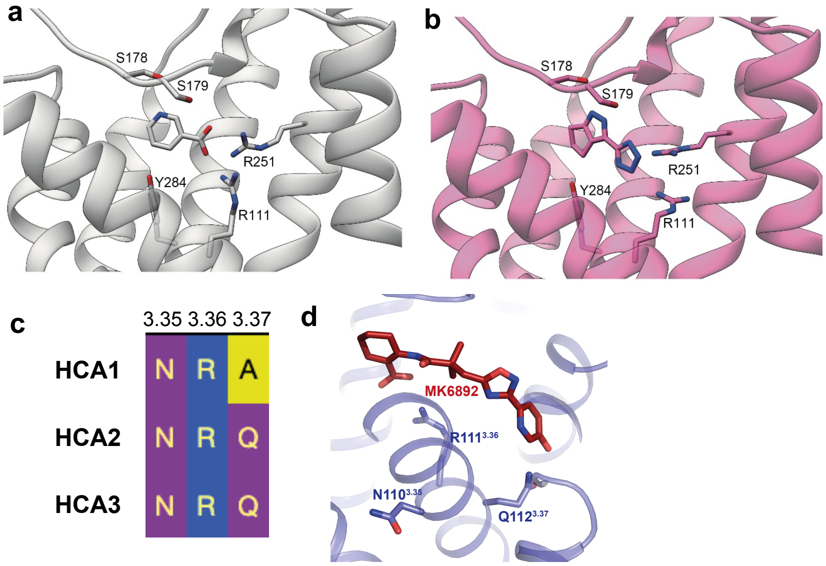

Supplementary Fig.10 Ligand recognition of HCA2 and sequence Sequence conservation of toggle switch in HCA receptor family.

(a) Docking of niacin with HCA2. **(b)** Docking of MK-0354 with HCA2. **(c)** Residues of 3.35-3.37 sequence alignment in Hydrocarboxylic acid receptor family. **(d)** Interaction of residue Q112 with MK-6892.

Supplementary Tables

Supplementary Table1. Summary of the model.

	Subunit	Chain	Total residues /Range built	Poly-ALA model	Ligand	Resolution (Å)
HCA2-G_i complex	HCA2	R	363/ 15-298	-	MK-6892	3.0-5.0
	Gα	A	356/ 5-54, 182-354	-	-	2.6-4.6
	Gβ	B	340/ 3-340	-	-	2.6-4.0
	Gγ	C	71/ 7-63	-	-	3.2-4.5
	scFv16	S	248/ 5-120, 136-245	-	-	2.8-4.0

Supplementary Table2. Stubbed residues in HCA2.

TMI	ICL1	TMII	ECL1	TMIII	ICL2	TMIV	ECL2	TMV	ICL3	TMVI	ECL3	TMVII
D23	L56	K60	-	-	-	-	-	-	-	-	-	-
F25	K57	I77										
I26	S58	I78										
L34	W59	D85										
L36		N86										
E37		V88										
F38												
I39												
L42												
L43												
N45												
L47												
L49												
I51												
F52												
C53												
H55												

Supplementary Table3. cAMP inhibition profile for mutants in the binding pocket as activated by MK-6892.

EC50 (pEC50± SEM) and Emax %±SEM were derived from n=3 experiments.

	**cAMP inhibition**	
Mutants	EC50, µM (pEC50±s.e.m)	Emax (%)

**WT**	0.05 (7.29±0.07)	100±4
**F277A**	0.11 (6.94±0.11)	43±3
**R111A**	N.D.	N.D.
**Q112A**	2.19 (5.66 ± 0.11)	26±8
**Q112E**	0.50 (6.30 ± 0.06)	56±5
**S287V**	N.D.	N.D.

Supplementary Table4. cAMP inhibition and β-arrestin activation profile for R111A and Q112A activated by MK-6892, niacin, MK-0354, MK-1903 and acifran.

EC50 (pEC50± SEM) and Emax %±SEM were derived from n=3 experiments.

		**WT**	**R111A**	**Q112A**
MK-6892	EC50, μM (pEC50±s.e.m)	0.05 (7.29±0.07)	N.D.	2.19 (5.66±0.11)
	fold difference	1	N.D	42
	Emax (%)	100±4	N.D.	26±8
niacin	EC50, μM (pEC50±s.e.m)	0.04 (7.37±0.08)	N.D.	0.21 (6.68±0.06)
	fold difference	1	N.D	4
	Emax (%)	100±2	N.D.	124±4
MK-0354	EC50, μM (pEC50±s.e.m)	0.69 (6.16±0.13)	N.D.	3.89
	fold difference	1	N.D	5
	Emax (%)	81±7	N.D.	11±5
MK-1903	EC50, μM (pEC50±s.e.m)	0.01 (7.94±0.09)	N.D.	0.038
	fold difference	1	N.D	3
	Emax (%)	90±4	N.D.	74±8
acifran	EC50, μM (pEC50±s.e.m)	0.31 (6.51±0.11)	N.D.	0.62
	fold difference	1	N.D	1.99
	Emax (%)	90±4	N.D.	26±7

Supplementary Table5. cAMP inhibition profile for 38 alanine mutations activated by MK-6892 and niacin. EC50 (pEC50± SEM) and Emax %±SEM were derived from n=3 experiments and log(Emax/EC50) or Δ log(Emax/EC50) or ΔΔlog(Emax/EC50) were calculated.

niacin						MK-6892						niacin-MK-6892
Mutants	EC50, μM (pEC50 ±s.e.m)	Emax (%)	log(Emax/EC50)	$\Delta\log(E_{max}/EC_{50})_{niacin}$	N	Mutants	EC50, μM (pEC50 ±s.e.m)	Emax (%)	log(Emax/EC50)	$\Delta\log(E_{max}/EC_{50})_{MK\text{-}6892}$	N	$\Delta\Delta\log(E_{max}/EC_{50})$ *

WT	0.04 (7.37±0.08)	100±2	7.37	0.0	6	WT	0.05 (7.29±0.07)	100±4	7.29	0.0	6	0.0
F193A	N.D.	N.D.	N.D.	N.D.	3	F193A	0.40 (6.40±0.11)	90±13	6.35	-0.9	3	N.D.
F277A	N.D.	N.D.	N.D.	N.D.	3	F277A	0.11 (6.94±0.11)	43±3	6.54	-0.8	3	N.D.
L280A	N.D.	N.D.	N.D.	N.D.	3	L280A	0.17 (6.76±0.10)	100±9	6.76	-0.5	3	N.D.
K165A	0.03 (7.53±0.12)	90±6	7.48	0.1	3	K165A	0.10 (7.00±0.13)	98±9	6.99	-0.3	3	0.4
K164A	0.04 (7.45±0.13)	76±6	7.33	0.0	3	K164A	0.07 (7.13±0.20)	77±10	7.02	-0.3	3	0.2
K166A	0.06(7.20±0.15)	73±7	7.06	-0.3	3	K166A	0.17 (6.78±0.15)	84±9	6.7	-0.6	3	0.3
F276A	N.D.	N.D.	N.D.	N.D.	3	F276A	0.71 (6.15±0.33)	60±39	5.93	-1.4	3	N.D.
R22A	0.11 (6.95±0.16)	100±11	6.95	-0.4	3	R22A	0.02 (7.70±0.08)	104±4	7.72	0.4	3	-0.9
Q264A	0.03 (7.47±0.15)	90±7	7.42	0.0	3	Q264A	0.08 (7.09±0.10)	102±7	7.1	-0.2	3	0.2
F180A	N.D.	N.D.	N.D.	N.D.	3	F180A	0.11 (6.96±0.09)	89±7	6.91	-0.4	3	N.D.
S181A	0.06 (7.23±0.11)	95±7	7.21	-0.2	3	S181A	0.01 (7.87±0.07)	111±4	7.92	0.6	3	-0.8
C18A	0.79 (6.10±0.65)	38±27	5.68	-1.7	3	C18A	0.02 (7.65±0.07)	118±4	7.72	0.4	3	-2.1
V20A	0.06 (7.25±0.16)	88±9	7.19	-0.2	3	V20A	0.03 (7.52±0.11)	94±6	7.49	0.2	3	-0.4
D92A	0.17 (6.76±0.25)	69±15	6.60	-0.8	4	D92A	0.03 (7.57±0.15)	98±8	7.56	0.3	4	-1.0
S178A	N.D.	N.D.	N.D.	N.D.	3	S178A	1.09 (5.96±0.09)	48±18	5.64	-1.7	3	N.D.
S179A	0.46 (6.34±0.14)	95±19	6.32	-1.1	3	S179A	0.01 (7.92±0.07)	133±5	8.04	0.7	3	-1.8
N86A	0.04 (7.39±0.12)	86±6	7.32	0.0	3	N86A	0.02 (7.67±0.13)	104±7	7.69	0.4	3	-0.5

Y87A	N.D.	N.D.	N.D.	N.D.	3	Y87A	0.02 (7.71±0.11)	122±7	7.8	0.5	3	N.D.
W91A	N.D.	N.D.	N.D.	N.D.	3	W91A	0.06 (7.22±0.11)	104±7	7.24	0.0	3	N.D.
H161A	0.11 (6.95±0.23)	83±13	6.87	-0.5	3	H161A	0.11 (6.97±0.18)	93±11	6.94	-0.4	3	-0.2
H189A	0.27 (6.57±0.15)	85±13	6.50	-0.9	3	H189A	0.048 (7.32±0.07)	115±4	7.38	0.1	3	-1.0
Y284A	N.D.	N.D.	N.D.	N.D.	3	Y284A	0.06 (7.21±0.08)	97±4	7.21	-0.1	3	N.D.
L107A	0.22 (6.65±0.37)	38±11	6.23	-1.1	3	L107A	0.16 (6.78±0.08)	99±5	6.78	-0.5	3	-0.6
R111A	N.D.	N.D.	N.D.	N.D.	3	R111A	N.D.	N.D.	N.D.	N.D.	3	N.D.
D73A	N.D.	N.D.	N.D.	N.D.	3	D73A	N.D.	N.D.	N.D.	N.D.	3	N.D.
D290A	0.35 (6.45±0.50)	30±20	5.93	-1.4	3	D290A	0.13 (6.88±0.15)	47±9	6.55	-0.7	3	-0.7
S114A	0.06 (7.22±0.20)	54±3	6.95	-0.4	3	S114A	1.12 (5.95±0.17)	46±4	5.61	-1.7	3	1.3
N286A	0.11 (6.95±0.23)	72±11	6.81	-0.6	3	N286A	0.44 (6.36±0.21)	60±18	6.14	-1.2	3	0.6
C19A	N.D.	N.D.	N.D.	N.D.	3	C19A	0.29 (6.54±0.15)	89±16	6.49	-0.8	3	N.D.
C100A	N.D.	N.D.	N.D.	N.D.	3	C100A	N.D.	N.D.	N.D.	N.D.	3	N.D.
C177A	N.D.	N.D.	N.D.	N.D.	3	C177A	N.D.	N.D.	N.D.	N.D.	3	N.D.
C183A	N.D.	N.D.	N.D.	N.D.	3	C183A	0.26 (6.59±0.19)	90±17	6.54	-0.8	3	N.D.
C266A	0.51 (6.29±0.1)	70±23	6.14	-1.2	3	C266A	0.09 (7.03±0.12)	108±9	7.06	-0.2	3	-1.0
F296A	0.08 (7.10±0.09)	157±10	7.30	-0.1	3	F296A	0.11 (6.96 ± 0.14)	166±16	7.18	-0.1	3	0.0
R128A	0.11 (6.95±0.13)	160±15	7.15	-0.2	3	R128A	0.05 (7.31 ± 0.12)	173±13	7.55	0.3	3	-0.5
A229G	0.23 (6.64±0.08)	169±3	6.87	-0.5	3	A229G	0.21 (6.68±0.14)	159±5	6.88	-0.4	3	-0.1
F232A	0.02 (7.65±0.08)	142±4	7.80	0.4	2	F232A	0.23 (6.63 ± 0.07)	92±5	6.59	-0.7	2	1.1

Q112A	0.21 (6.68±0.06)	124±4	6.77	-0.6	3	Q112A	2.19 (5.66 ± 0.11)	26±8	5.07	-2.2	3	1.6

Supplementary References

57. Tate CG. A crystal clear solution for determining G-protein-coupled receptor structures. Trends in biochemical sciences. 2012;37(9):343-52.

58. Kabsch W. Xds. Acta Crystallogr D Biol Crystallogr. 2010;66(Pt 2):125-32.

59. Collaborative Computational Project N. The CCP4 suite: programs for protein crystallography. Acta Crystallogr D Biol Crystallogr. 1994;50(Pt 5):760-3.

60. McCoy AJ, Grosse-Kunstleve RW, Adams PD, Winn MD, Storoni LC, Read RJ. Phaser crystallographic software. J Appl Crystallogr. 2007;40(Pt 4):658-74.

61. Adams PD, Afonine PV, Bunkoczi G, Chen VB, Davis IW, Echols N, et al. PHENIX: a comprehensive Python-based system for macromolecular structure solution. Acta Crystallogr D Biol Crystallogr. 2010;66(Pt 2):213-21.

62. Smart OS, Womack TO, Flensburg C, Keller P, Paciorek W, Sharff A, et al. Exploiting structure similarity in refinement: automated NCS and target-structure restraints in BUSTER. Acta Crystallogr D Biol Crystallogr. 2012;68(Pt 4):368-80.

63. Emsley P, Lohkamp B, Scott WG, Cowtan K. Features and development of Coot. Acta Crystallogr D Biol Crystallogr. 2010;66(Pt 4):486-501.

64. Hua T, Li X, Wu L, Iliopoulos-Tsoutsouvas C, Wang Y, Wu M, et al. Activation and Signaling Mechanism Revealed by Cannabinoid Receptor-G(i) Complex Structures. Cell. 2020;180(4):655-65 e18.

65. Mastronarde DN. Automated electron microscope tomography using robust prediction of specimen movements. J Struct Biol. 2005;152(1):36-51.

66. Zheng SQ, Palovcak E, Armache JP, Verba KA, Cheng Y, Agard DA. MotionCor2: anisotropic correction of beam-induced motion for improved cryo-electron microscopy. Nat Methods. 2017;14(4):331-2.

67. Zhang K. Gctf: Real-time CTF determination and correction. J Struct Biol. 2016;193(1):1-12.

68. Punjani A, Rubinstein JL, Fleet DJ, Brubaker MA. cryoSPARC: algorithms for rapid unsupervised cryo-EM structure determination. Nat Methods. 2017;14(3):290-6.

69. Zivanov J, Nakane T, Scheres SHW. A Bayesian approach to beam-induced motion correction in cryo-EM single-particle analysis. IUCrJ. 2019;6(Pt 1):5-17.

70. Pettersen EF, Goddard TD, Huang CC, Couch GS, Greenblatt DM, Meng EC, et al. UCSF Chimera--a visualization system for exploratory research and analysis. J Comput Chem. 2004;25(13):1605-12.

71. Kelley LA, Mezulis S, Yates CM, Wass MN, Sternberg MJ. The Phyre2 web portal for protein modeling, prediction and analysis. Nat Protoc. 2015;10(6):845-58.

72. Kroeze WK, Sassano MF, Huang XP, Lansu K, McCorvy JD, Giguere PM, et al. PRESTO-Tango as an open-source resource for interrogation of the druggable human GPCRome. Nat Struct Mol Biol. 2015;22(5):362-9.
