# Supplementary material for: Structural insights into the human niacin receptor HCA2-Gi signalling complex
Source: Nat Commun. 2023 Mar 27;14:1692. doi: 10.1038/s41467-023-37177-6 (PMC10043007; doi:10.1038/s41467-023-37177-6)
Supplement: Supplementary file 2 — Reporting Summary [file 41467_2023_37177_MOESM2_ESM.pdf]

nature portfolio

Corresponding author(s): Wei Peng, Bryan L. Roth, Zihe Rao and Zhijie Liu

Last updated by author(s): Feb 3, 2023

Reporting Summary

Nature Portfolio wishes to improve the reproducibility of the work that we publish. This form provides structure for consistency and transparency in reporting. For further information on Nature Portfolio policies, see our Editorial Policies and the Editorial Policy Checklist.

Statistics

For all statistical analyses, confirm that the following items are present in the figure legend, table legend, main text, or Methods section.

n/a	Confirmed	
☐	☒	The exact sample size (*n*) for each experimental group/condition, given as a discrete number and unit of measurement
☐	☒	A statement on whether measurements were taken from distinct samples or whether the same sample was measured repeatedly
☒	☐	The statistical test(s) used AND whether they are one- or two-sided<br>*Only common tests should be described solely by name; describe more complex techniques in the Methods section.*
☒	☐	A description of all covariates tested
☒	☐	A description of any assumptions or corrections, such as tests of normality and adjustment for multiple comparisons
☐	☒	A full description of the statistical parameters including central tendency (e.g. means) or other basic estimates (e.g. regression coefficient) AND variation (e.g. standard deviation) or associated estimates of uncertainty (e.g. confidence intervals)
☒	☐	For null hypothesis testing, the test statistic (e.g. *F*, *t*, *r*) with confidence intervals, effect sizes, degrees of freedom and *P* value noted<br>*Give P values as exact values whenever suitable.*
☒	☐	For Bayesian analysis, information on the choice of priors and Markov chain Monte Carlo settings
☒	☐	For hierarchical and complex designs, identification of the appropriate level for tests and full reporting of outcomes
☒	☐	Estimates of effect sizes (e.g. Cohen's *d*, Pearson's *r*), indicating how they were calculated

Our web collection on statistics for biologists contains articles on many of the points above.

Software and code

Policy information about availability of computer code

Data collection	Cryo-EM single particle data was automatically collected on the Titan Krios using serialEM 3.7.3.
Data analysis	XDS v2017, Buster V2.10.1, GraphPad Prism v5.1 and v8.1, Phenix v1.18.2, COOT v0.9.6, cryoSPARC v3.3.0, RELION v3.1, Phyre2, ChimeraX v1.2.5 , Refmac5 v5.8, Schrödinger Suites 2018-2, PyMol 2.4.1, ChemDraw 20.0.0

For manuscripts utilizing custom algorithms or software that are central to the research but not yet described in published literature, software must be made available to editors and reviewers. We strongly encourage code deposition in a community repository (e.g. GitHub). See the Nature Portfolio guidelines for submitting code & software for further information.

Data

Policy information about availability of data

All manuscripts must include a data availability statement. This statement should provide the following information, where applicable:

- Accession codes, unique identifiers, or web links for publicly available datasets
- A description of any restrictions on data availability
- For clinical datasets or third party data, please ensure that the statement adheres to our policy

The atomic coordinates for HCA2-3378, HCA2-8519 and HCA2-MK6892-Gi-scFv16 have been deposited in the Protein Data Bank with the accession codes 7ZLY, 7ZL9 and 7XK2. The EM maps for HCA2-MK6892-Gi-scFv16 have been deposited in EMDB with the codes EMD-33241.

Human research participants

Policy information about studies involving human research participants and Sex and Gender in Research.

Reporting on sex and gender	n/a
Population characteristics	n/a
Recruitment	n/a
Ethics oversight	n/a

Note that full information on the approval of the study protocol must also be provided in the manuscript.

Field-specific reporting

Please select the one below that is the best fit for your research. If you are not sure, read the appropriate sections before making your selection.

☒ Life sciences ☐ Behavioural & social sciences ☐ Ecological, evolutionary & environmental sciences

For a reference copy of the document with all sections, see nature.com/documents/nr-reporting-summary-flat.pdf

Life sciences study design

All studies must disclose on these points even when the disclosure is negative.

Sample size	No statistical methods used to predetermine sample size. For cryoEM studies, the number of micrographs is determined by the available microscope time. For the functional cell based assays, three biologically independent experiments (n=3) were performed.
Data exclusions	No data were excluded.
Replication	At least three independent biological repeats per experiment where representative data is shown. Cell-based signaling assays were independently replicated by two investigators. All attempts at replication were successful.
Randomization	Drug treatments were performed in dose-response studies on the same set of cells that also received control treatments and vehicle treatments on the same plates. All normalization to control and baseline/vehicle occurred within plate, then averaged among replicates. Drug treatment was randomized on the plate to avoid "plate effects." Wild-type controls were always run in parallel with mutant receptors.
Blinding	No blinding was performed in this study. For both cryoEM, x-ray structure determination and functional studies, blinding is not necessary due to the nature of these experiments do not requires subject assessment of the data that may influence the validity of the results.

Reporting for specific materials, systems and methods

We require information from authors about some types of materials, experimental systems and methods used in many studies. Here, indicate whether each material, system or method listed is relevant to your study. If you are not sure if a list item applies to your research, read the appropriate section before selecting a response.

Materials & experimental systems

n/a	Involved in the study	
☐	☒	Antibodies
☐	☒	Eukaryotic cell lines
☒	☐	Palaeontology and archaeology
☒	☐	Animals and other organisms
☒	☐	Clinical data
☒	☐	Dual use research of concern

Methods

n/a	Involved in the study	
☒	☐	ChIP-seq
☒	☐	Flow cytometry
☒	☐	MRI-based neuroimaging

Antibodies

Antibodies used	Antibody used was presented in the Methods section with validation referenced. Anti-FLAG–horseradish peroxidase–conjugated antibody (Sigma-Aldrich, A8592, clone M2) diluted 1/10,000.

Validation	Anti-FLAG–horseradish peroxidase–conjugated antibody is from mouse clone M2 and used for measuring protein expression on the surface of cells. Detailed information can be found at: https://www.sigmaaldrich.com/US/en/product/sigma/a8592

Eukaryotic cell lines

Policy information about cell lines and Sex and Gender in Research

Cell line source(s)	Spodoptera frugiperda (Sf9) cells were a gift from Dr. Beili Wu (SIMM, CAS). HEK293T cells were purchased from the American Type Culture Collection (ATCC, ATCC CRL-11268). HTLA cells (an HEK293 cell line stably expressing a tTA-dependent luciferase reporter and a -arrestin2-TEV fusion gene) were generously provided by Richard Axel's lab.
Authentication	Sf9 cells were a gift from Dr. Beili Wu and was authenticated for protein expression. HEK293T cells were authenticated by the supplier (ATCC) using morphology and growth characteristics, and STR profiling.HEK293 cells were authenticated using morphologies and growth characteristics according to instructions on ThermoFisher website. HTLA cells (a HEK293 cell line stably expressing a tTA-dependent luciferase reporter and a β-arrestin2-TEV fusion gene) were a gift from R. Axel and was authenticated by morphology, growth characteristics and the successful tango assay which demonstrates that both tTA-dependent luciferase reporter and a β-arrestin2-TEV fusion gene are presented in the cells.
Mycoplasma contamination	All cells have been tested as negative for mycoplasma contamination.
Commonly misidentified lines (See ICLAC register)	No commonly misidentified cell lines were used.
